# Risk factors for unavoidable removal of instrumentation after surgical site infection of spine surgery: A retrospective case-control study

**DOI:** 10.1097/MD.0000000000005118

**Published:** 2016-10-28

**Authors:** Hiroyuki Tominaga, Takao Setoguchi, Hideki Kawamura, Ichiro Kawamura, Satoshi Nagano, Masahiko Abematsu, Fumito Tanabe, Yasuhiro Ishidou, Takuya Yamamoto, Setsuro Komiya

**Affiliations:** a Department of Orthopaedic Surgery, Graduate School of Medical and Dental Sciences; b Near-Future Locomotor Organ Medicine Creation Course (Kusunoki Kai), Graduate School of Medical and Dental Sciences, Kagoshima University; c Division of Medical and Environmental Safety, Kagoshima University Medical and Dental Hospital; d Medical Joint Materials, Graduate School of Medical and Dental Sciences, Kagoshima University, Sakuragaoka; e Yonemori Hospital, Yojiro, Kagoshima, Japan.

**Keywords:** instrumentation, spine surgery, surgical site infection, unavoidable removal

## Abstract

Surgical site infection (SSI) after spine instrumentation is difficult to treat, and often requires removal of instrumentation. The removal of instrumentation after spine surgery is a severe complication that can lead to the deterioration of activities of daily living and poor prognosis. Although there are many reports on SSI after spine surgery, few reports have investigated the risk factors for the removal of instrumentation after spine surgery SSI. This study aimed to identify the risk factors for unavoidable removal of instrumentation after SSI of spine surgery. We retrospectively reviewed 511 patients who underwent spine surgery with instrumentation at Kagoshima University Hospital from January 2006 to December 2014. Risk factors associated with SSI were analyzed via multiple logistic regression analysis. Parameters of the group that needed instrumentation removal were compared with the group that did not require instrumentation removal using the Mann–Whitney *U* and Fisher's exact tests. The posterior approach was used in most cases (453 of 511 cases, 88.6%). SSI occurred in 16 of 511 cases (3.14%) of spine surgery with instrumentation. Multivariate logistic regression analysis identified 2 significant risk factors for SSI: operation time, and American Society of Anesthesiologists physical status classification ≥ 3. Twelve of the 16 patients with SSI (75%) were able to keep the instrumentation after SSI. Pseudarthrosis occurred in 2 of 4 cases (50%) after instrumentation removal. Risk factors identified for instrumentation removal after spine SSI were a greater number of past surgeries, low preoperative hemoglobin, high preoperative creatinine, high postoperative infection treatment score for the spine, and the presence of methicillin-resistant *Staphylococcus aureus*. In these high risk cases, attempts should be made to decrease the risk factors preoperatively, and careful postoperative monitoring should be conducted.

## Abstract 2

Supplemental Digital Content is available in the text

## 1 Introduction

Surgical site infection (SSI) after spine surgery has serious consequences^[1]^; it extends the period in which the patient is bedridden and increases mortality.^[2]^ SSI after spine instrumentation surgery is especially difficult to treat, and often leads to removal of instrumentation to treat the infection. Removal of instrumentation after spine surgery is associated with severe complications such as pseudarthrosis, which results in a deterioration of the activities of daily living and a poor prognosis.

There are many reports on SSI after spine surgery; risk factors for SSI after spine surgery include obesity, longer operation time, diabetes mellitus, and smoking.^[^3–6^]^ However, there are few reports regarding risk factors for removal of instrumentation after spine surgery.^[^7–9^]^ We aimed to identify the risk factors for removal of instrumentation after SSI after spine surgery and to investigate the prognosis of these cases.

## 2 Materials and methods

We retrospectively reviewed 511 patients who underwent spinal instrumentation surgery at Kagoshima University Hospital from January 2006 to December 2014. We excluded patients who had undergone instrumentation removal after achievement of bone union and those who had undergone external skeletal fixation with a halo vest.

Risk factors for spinal SSIs with instrumentation were analyzed via multiple logistic regression analysis. The parameters of the patients with instrumentation removal were compared with the parameters of those without removal. The Mann–Whitney *U*-test was used for numerical data (patient age, operation time, blood loss, white blood cell count [WBC], and body mass index [BMI]). Fisher's exact probability test was used to identify differences in the expected versus the observed frequency of nominal variables (sex, diabetes mellitus, pathogenic bacteria, and sepsis). *P* < 0.05 was considered statistically significant. The software used for analyses was BellCurve for Excel (Social Survey Research Information Co., Ltd. Tokyo, Japan), which is add-in software to Excel for statistical evaluation.

Instrumentation was removed in cases of uncontrollable infection or fixation loosening after wound irrigation and debridement. Postoperative infection treatment score for the spine (PITSS) was measured as described by Dipaola et al.^[10]^ Measures were undertaken preoperatively to prevent SSI in cooperation with an infection surveillance team; before surgery, we ensured that patients had HbA1C <7.0%, hemoglobin (Hb) >11.0 g/dL, steroid ≤5 mg, total protein (TP) > 6.0 g/dL, and had not smoked for ≥4 weeks, except in 1 case of an emergency operation due to paralysis. In conjunction with our infection control team, we preoperatively detected any methicillin-resistant *Staphylococcus aureus* (MRSA) carriers using nasal swabs. We followed the Center for Disease Control (CDC) guidelines, and defined any infection of the surgical incision that occurred in the first 90 postoperative days as an SSI. All patients had >1 year of follow-up.

Urinary tract infection (UTI) was diagnosed when there were ≥5 white blood cells per high-power field in unspun urine combined with the presence of at least 2 signs or symptoms of UTI (fever, polyuria, dysuria, or suprapubic tenderness).^[11]^ Sepsis was diagnosed by blood culture and the presence of fever .^[12]^

Patients were treated according to the CDC guidelines for preventing SSI.^[13]^ Since 2009, our protocol has been to administer 1 to 2 g of cefazolin (according to the appropriate dose for the patient's weight) 30 min before skin incision, and then every 3 hours during surgery, and again if blood loss exceeds 1000 mL during the first 24 hours following wound closure. Antibiotic prophylaxis is conducted for at least 2 days after surgery, and we thoroughly sterilize our fingers with alcohol to avoid contact infection.^[14]^ The local ethics committee of Kagoshima University reviewed and approved this study, and no specific funding was obtained.

## 3 Results

The median patient age was 57.0 years (range 18–70 years), and 234 of the 511 patients were males (45.8%). Cases of pyogenic spondylitis, tumor, and scoliosis involved the cervical, thoracic, and lumbosacral spine. SSI after spine instrumentation occurred in 16 of 511 cases (3.14%). The posterior approach was used in 453 of 511 cases (88.6%, Table 1). The median number of posterior fusion levels was 3 (range 2–7), median number of anterior fusion levels was 3.5 (range 2–4), and median number of anterior and posterior fusion levels was anterior 2 (range 1–3.5) and posterior 4 (range 2–5). SSI did not occur in any case that used anterior fusion (Table 1). There were 177 cases of scoliosis and the median number of posterior fusion levels of scoliosis was 7 (range 4–11).

**Table 1:**
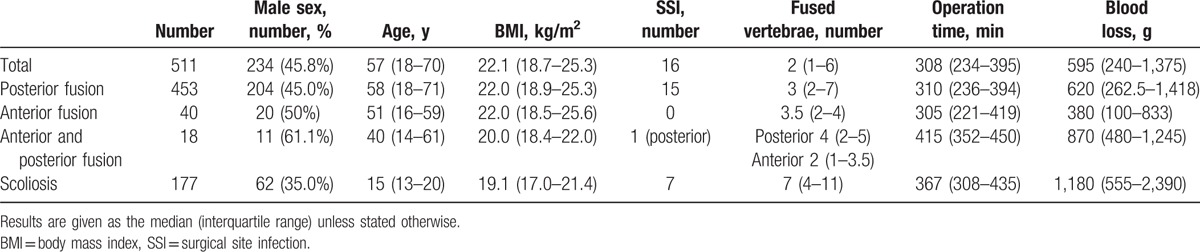
Details of cases of spine fusion with instrumentation.

The patients with ≥5 fused segments had a significantly higher incidence of scoliosis and were significantly younger than the patients with <5 fused segments (both *P* < 0.001, Supplemental Table 1). Multiple logistic regression analysis indicated that the common risk factors for SSI were operation time (HR 1.007, 95% CI 1.003–1.011, *P* = 0.0014) and ASA classification ≥3 (HR 5.3, 95% CI 1.4–19.9, *P* = 0.014, Supplemental Table 2).

Instrumentation removal was avoided in 12 of the 16 SSI cases (75%) (Table 2). The median time from surgery to the onset of SSI was 14.5 days (range 11.0–21.3 days); the median PITSS was 22 (range 18–24). The bacteria causing SSI were MRSA in 4 cases, and multipathogenic bacteria in 5 cases. Supplemental Table 3 contains the details of cases in which instrumentation had to be removed following surgical site infection after spine surgery. The primary operation was performed in another hospital other than case2.

**Table 2:**
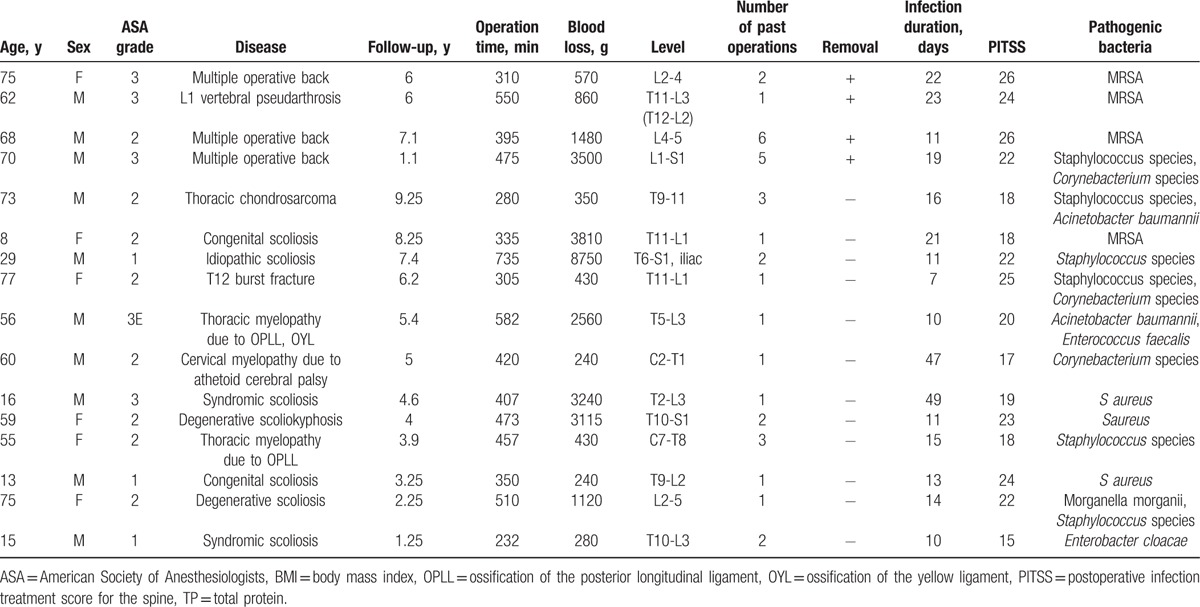
Details of surgical site infection cases with instrumentation.

The Mann–Whitney *U*-test and Fisher's exact probability test identified the following as factors significantly associated with instrumentation removal after SSI: greater number of past surgeries, low preoperative Hb, high preoperative Cr, high PITSS, and the presence of MRSA (Table 3).

**Table 3:**
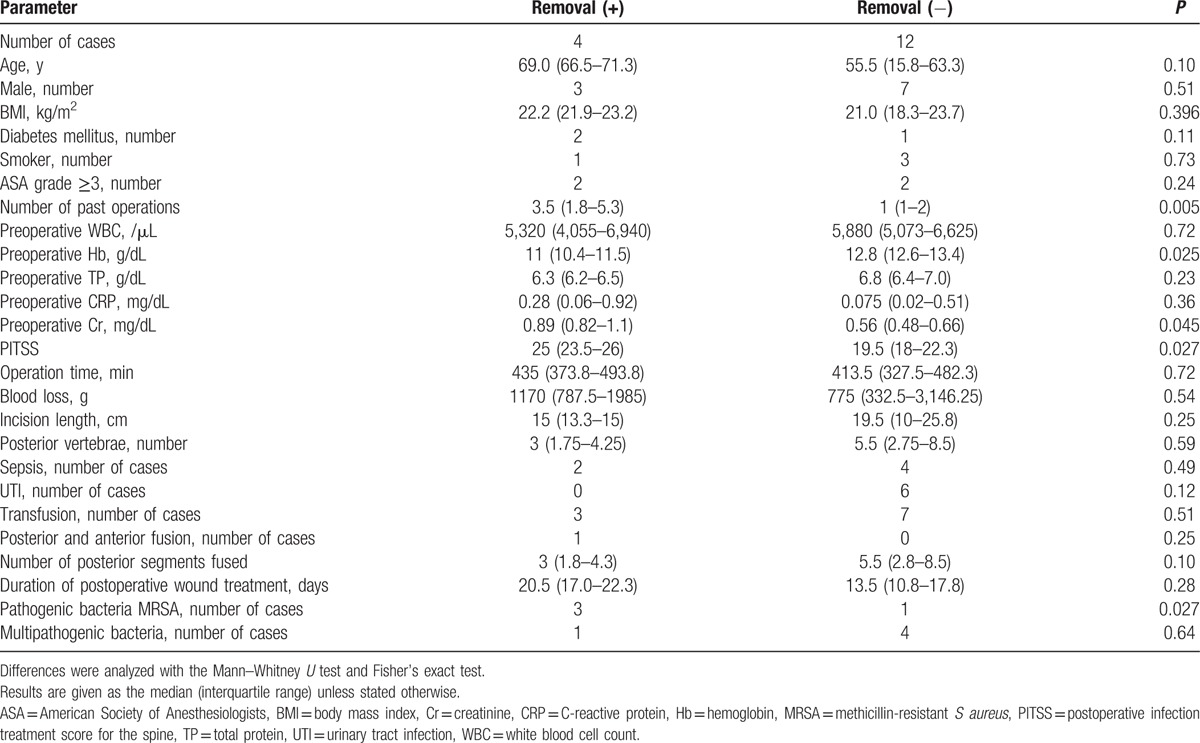
Comparison of spinal surgical site infection cases that required instrumentation removal with those that did not.

Two of the four cases (50%) requiring instrumentation removal resulted in pseudarthrosis. The signs of infection calmed down within 1 month of instrumentation removal, but both patients died 6 years after the operation because of renal failure. The cases without instrumentation removal did not result in pseudarthrosis, but 2 cases underwent additional surgery because of adjacent segmental disease. We started a new protocol in 2009, and the instrumentation preservation rate has since improved to 9/10 after spinal SSI with instrumentation, and the rate of MRSA as the pathogenic bacteria was also improved (Table 4).

**Table 4:**
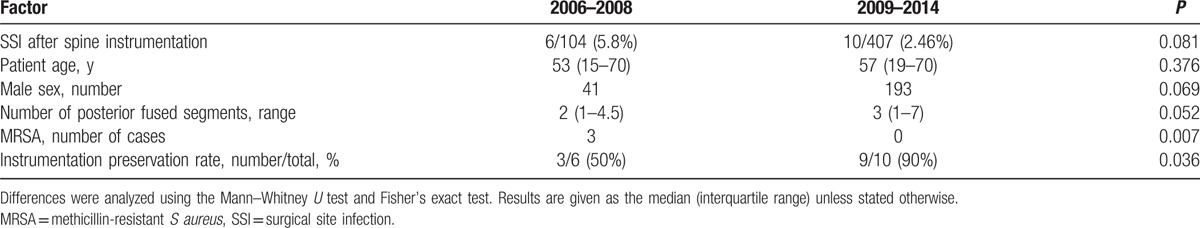
Comparison of spine surgical site infection parameters before and after implementation of our infection reduction protocol in 2009.

## 4 Discussion

In this study, the risk factors for SSI after spine surgery were longer operation time and ASA grade ≥3. The risk of unavoidable instrumentation removal after SSI was significantly increased if patients had undergone a greater number of past surgeries, had low preoperative Hb, high preoperative Cr, high PITSS, and in cases where MRSA was present.

Previous studies have reported the risk factors for SSI in patients who have undergone spine surgery; there is strong evidence that the independent risk factors are obesity, longer operation time, diabetes mellitus, smoking, history of previous SSI, and type of surgical procedure.^[^3–6^]^ In the present study, common risk factors for SSI were longer operation time and ASA classification ≥3, consistent with other reports.^[^4–6^]^ Renal disease was identified as a risk factor for SSI in a previous regression analysis of 1532 patients.^[16]^ Renal failure leads to immunodeficiency, and the weakness of bones might affect SSI. Low preoperative Hb level is another risk factor for SSI,^[17]^ and preoperative correction of Hb may reduce the likelihood of postoperative SSI. Patients who had undergone a previous spinal surgery are at high risk for infection compared with those with no prior surgical history.^[18]^ Multiple back surgeries may lead to poor soft tissue cover and a poor blood supply, which may prevent wound healing. Cizik et al^[16]^ reported that diabetes mellitus was significantly associated with SSI after spine surgery. In contrast, we found that diabetes mellitus was not a risk factor associated with SSI after spine surgery. This difference may be because all patients in our study had preoperative HbA1C < 7.0%, while the patients in the study by Cizik et al^[16]^ may have had more severe diabetes mellitus.

Several previous studies have investigated risk factors for treatment failure after spine SSI. Maruo and Beven^[7]^ reported lower treatment success rates after spine SSI in cases involving late infection, fusion with fixation to the ilium, *Propionibacterium acnes*, polymicrobial infection, >6 operated spinal levels, and instrumentation; late infection was the most significant independent risk factor associated with treatment failure.^[7]^ Kowalski et al^[15]^ reported that the presence of pre-existing malignancy or radiation therapy were significant risk factors for treatment failure.^[15]^ Núñez-Pereira et al^[8]^ reported that 8.9% of patients treated with posterior spinal fusion and instrumentation had a deep SSI; multivariate analysis revealed a significant risk of treatment failure in patients who developed sepsis or who had >3 fused segments.^[8]^ In contrast, we found that a higher number of fused segments was not a risk factor for treatment failure, which was defined as implant removal after SSI. One potential reason for this difference is that the patients in our study with ≥5 fused segments had a significantly higher incidence of scoliosis and were significantly younger than the patients with <5 fused segments. Dipaola et al^[10]^ reported that PITSS was a predictor of risk for multiple irrigation and debridement after spinal SSI. In the present study, risk factors for removal of instrumentation after spine surgery were: greater number of past operations, low preoperative Hb, high preoperative Cr, high PITSS, and the presence of MRSA. Our findings suggest that PITSS may be an important predictor of instrumentation removal after spine SSI.

To prevent MRSA infection, we previously reported that the application of vancomycin-impregnated fibrin sealant to spinal instrumentation yielded good clinical outcomes regarding the prevention of postoperative spinal infections.^[19]^ Subsequently, it has been reported that vancomycin administration to the operation field reduces the overall costs after SSI with instrumentation.^[20]^ However, the FDA has not currently approved vancomycin as an intrawound application, because a well-designed prospective study has not yet been conducted. ^[21]^ The MRSA infection rate is negatively correlated with both the density of cefazolin antimicrobial use and the use of an alcohol antiseptic agent.^[14]^ Hence, in 2009, we implemented a protocol of 48 hours of prophylactic antimicrobial agent administration and cefazolin, and an increase in the quantity of thorough hand washing with alcohol; since then, the rate of MRSA infection in our institution has decreased (SSI rate: 10 of 407 spine surgery cases, MRSA rate: zero of 10 SSI cases).

The reported rate of pseudarthrosis after spine surgery is 37.9%,^[22]^ and there is a 71% 2-year cumulative probability of treatment failure-free survival after SSI.^[15]^ In the present study, the pseudarthrosis rate after instrumentation removal was 50% (2 of 4 cases). Since we started a new protocol in 2009, the instrumentation preservation rate has improved to 9 out of 10 cases after SSI of spinal instrumentation surgery and the pseudarthrosis rate after SSI is now 0%.

There are no clear predictors of whether we can safely reinsert instrumentation after SSI. Currently, we perform reinsertion of instrumentation after SSI if there are no signs of infection of vertebrae and disk on magnetic resonance imaging, no indicators of infection on blood test results, and <5 polymorphonuclear leukocyte cells/high power field in intraoperative pathological examination.^[23]^

This study had some limitations. First, the number of included patients was relatively small. Second, it was a retrospective study. Finally, the diagnosis and surgery types varied in the 4 cases that required instrumentation removal, and also varied in the 12 cases that did not require instrumentation removal.

In conclusion, risk factors for removal of instrumentation after spine surgery were: greater number of past operations, low preoperative Hb, high preoperative Cr, high PITSS, and the presence of MRSA. Surgeons should perform spine surgery after implementing the abovementioned precautionary measures to limit postoperative complications.

## Acknowledgments

The authors thank Ms. Ayano Komure, Ms. Rika Sakamoto, and Ms. Kana Maeda for their excellent assistance.

## Supplementary Material

Supplemental Digital Content
