# Supplementary material for: Risk factors for unavoidable removal of instrumentation after surgical site infection of spine surgery: A retrospective case-control study
Source: Medicine (Baltimore). 2016 Oct 28;95(43):e5118. doi: 10.1097/MD.0000000000005118 (PMC5089094; doi:10.1097/MD.0000000000005118)
Supplement: Supplemental Digital Content [file medi-95-e5118-s001.docx]

Supplemental Table 1 Relationship of age and scoliosis with number of posterior segments fused

| Number of posterior segments fused | ≥ 5 | < 5 | *P* value |
| --- | --- | --- | --- |
| Age | 19 (14–58) | 62 (42–72) | < .001 |
| Scoliosis | 118 | 59 | < .001 |

Results are given as the median (interquartile range).

Differences were analyzed with the Mann-Whitney U test and Fisher’s exact test.

Supplemental Table 2 Risk factors for surgical site infection identified by multivariate logistic regression analysis

| **Parameter** | **Univariate analysis** | | **Multivariate analysis** | |
| --- | --- | --- | --- | --- |
|  | OR (95% CI) | *P* value | OR (95% CI) | *P* value |
| Age | 1.005 (0.99–1.03) | .63 | - | |
| Female sex | 0.50 (0.18–1.40) | .19 | - | |
| ASA grade ≥ 3 | 6.02 (1.78–20.3) | **.004** | 5.3 (1.4–19.9) | **.014** |
| Diabetes mellitus | 1.17 (0.32–4.21) | .81 | - | |
| Smoking | 1.41 (0.44–4.50) | .56 | - | |
| BMI | 0.98 (0.89–1.09) | .73 | - | |
| Number of past operations | 1.34 (0.96–1.86) | .09 | 1.35 (0.89–2.05) | .15 |
| Preoperative TP | 0.54 (0.23–1.26) | .16 | 0.6 (0.24–1.48) | .26 |
| Operation time | 1.007 (1.003–1.011) | **< .001** | 1.007 (1.003–1.011) | **.0014** |
| Blood loss | 1.000 (1.000–1.0001) | .63 | - | |
| Transfusion | 0.77 (0.27–2.16) | .62 | - | |

ASA: American Society of Anesthesiologists, BMI: body mass index, TP: total protein, OR: odds ratio, CI: confidence interval.

Supplemental Table 3

Details of cases involving unavoidable instrumentation removal following surgical site infection after spine surgery

| Case | Age (years) | Sex | Disease |  | Operation | I&D | Loosening |
| --- | --- | --- | --- | --- | --- | --- | --- |
| 1 | 75 | F | L3/4 LDH | 1^st^ | decompression | 1 | + (L2) |
|  |  |  | L3 vertebral fracture | 2^nd^ | fusion (L2-4) |  |  |
| 2 | 62 | M | L1 vertebral pseudoarthrosis | 1^st^ | fusion (T11-L3) | 2 | - |
| 3 | 68 | M | L4/5 LSS | 1^st^ | Decompression | > 2  skin flap | - |
|  |  |  | L4 spondylolisthesis | 2^nd^ | fusion (L4-5) |  |  |
|  |  |  | L3/4 LSS | 3^rd^ | decompression (dural tear) |  |  |
|  |  |  | L3/4 dural tear | 4^th^ | 1^st^ dural repair |  |  |
|  |  |  | L3/4 dural tear | 5^th^ | 2^nd^ dural repair |  |  |
|  |  |  | L4/5 pseudoarthrosis | 6^th^ | refusion (L4-5) |  |  |
| 4 | 70 | M | L2/3 LSS | 1^st^ | decompression | 2 | + (L1) |
|  |  |  | L3/4 LDH | 2^nd^ | decompression |  |  |
|  |  |  | L4/5 LDH | 3^rd^ | decompression |  |  |
|  |  |  | L2/3, L3/4 LDH recurrence, L5/S1 LDH, L4 spondylolisthesis | 4^th^ | fusion (L2-S1) |  |  |
|  |  |  | L1/2 adjacent segmental  L2/3, L5/S1 nonunion | 5^th^ | fusion（L1-S1） |  |  |

I&D: irrigation and debridement (number of times); LDH: lumbar disc herniation; LSS: lumbar spinal stenosis
